# Acquired Bilateral Nevus of Ota-Like Macules: A Case Report

**DOI:** 10.7759/cureus.65453

**Published:** 2024-07-26

**Authors:** Jesús Iván Martínez-Ortega, Samantha Franco González, Ilse Fernández-Reyna

**Affiliations:** 1 Dermatology, Jalisco Dermatology Institute, Zapopan, MEX; 2 Internal Medicine, National Medical Center 21st Century, Mexico City, MEX; 3 Mycology, Yucatan Dermatological Center, Mérida, MEX

**Keywords:** cellular senescence, hori's nevus, melanoma risk, scleral involvement, blue pigmentation, q-switched laser therapy, acquired bilateral nevus of ota-like macules, ota nevus, dermal melanocytoses, facial melanoses

## Abstract

Facial melanoses (FM) present complex diagnostic and therapeutic challenges, particularly in the setting of dermal melanocytoses (DM). We present a case that illustrates these challenges as it does not fit within existing classification frameworks. Initially considered as Ota nevus, characterized by blue or dark pigmentation and scleral involvement, histopathological findings suggested acquired bilateral nevus of Ota-like macules (ABNOM). While ABNOM, more common in Asians, rarely affects the sclera or children, recent studies indicate that it may be underdiagnosed in these groups. Differential diagnosis ruled out other FM causes due to mucosal involvement. Correct classification is essential for epidemiological accuracy and treatment decisions, especially given varying responses to Q-switched laser therapy and melanoma risks associated with Ota nevus and ABNOM. While the pathogenesis remains unclear, a two-hit model involving shared melanoma mutations in melanocytes has been proposed and warrants further molecular study.

## Introduction

Facial melanoses (FM) is associated with complex diagnostic and therapeutic challenges, involving several well-defined clinical entities. Some forms of FM are caused by dermal melanocytoses (DM), which share overlapping features and are often difficult to classify [1,2]. We report a case that sheds light on these challenges, as it does not fit within the existing epidemiological or classification frameworks.

## Case presentation

**Figure 1:**
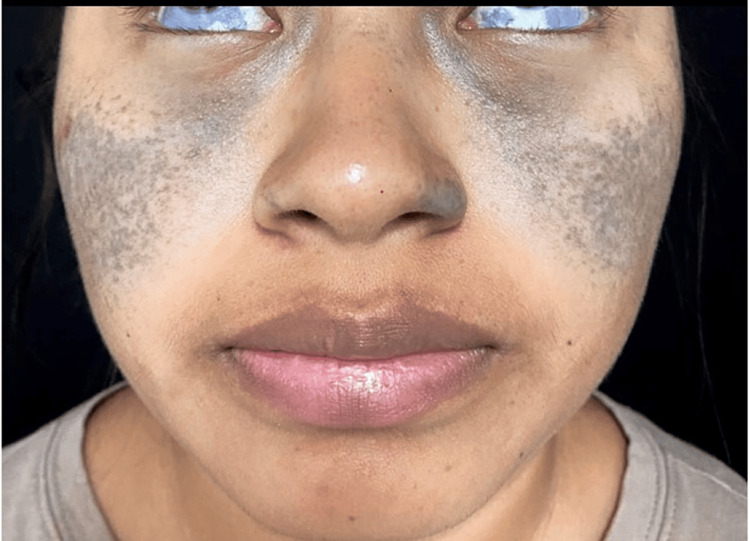
Clinical presentation of the patient Note the conjunctival affection, along with the irregular, confluent, bilateral, and symmetrical blue-violet macules on the face

An indigenous (Maya) 20-year-old female from Yucatan, Mexico, presented with a facial spot since the age of eight years. She denied any hearing loss, visual impairment, or other significant medical history. There was no use of hydroquinone, cosmetics, or systemic drugs, and no family history of abnormal cutaneous pigmentation. The patient was a housewife, married, and had one child. She had not received any previous treatments for her condition. On physical examination, a dermatosis was observed on the face, predominantly in the malar area and nasal wings, with the involvement of the lower bulbar conjunctiva, and characterized by irregular, confluent, bilateral, and symmetrical blue-violet macules; there was no involvement of the oral mucosa, tympanic membrane, or palmoplantar, no oral mucosal affection (Figure 1).

**Figure 2:**
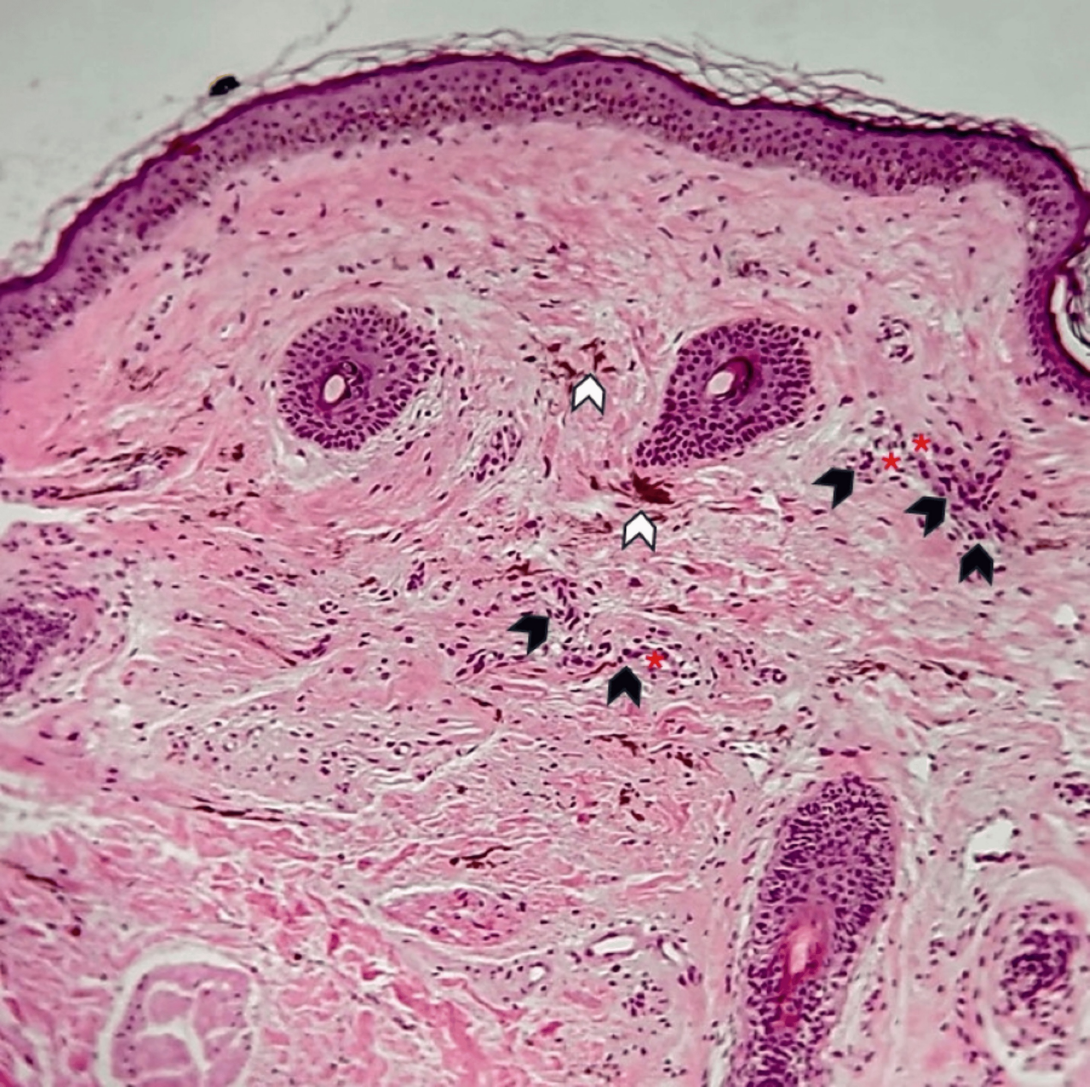
Biopsy A hematoxylin and eosin micrograph at 100x magnification shows clusters of melanocytes (black arrowheads) around and near vessels (red asterisks) and melanophages (white arrowheads) in the dermis

Our primary differential diagnosis was Ota nevus. To confirm this, we performed a biopsy, which revealed dendritic and fusiform melanocytes in the papillary dermis, diffusely infiltrating the collagen interstitium. Additionally, melanophages extending into the reticular dermis were observed, confirming the bilateral presentation of Ota nevus based on the pathological report (Figure 2). Unfortunately, our public dermatology center does not have access to a Q-switched laser. We recommended that the patient seek treatment at a facility equipped with this technology. However, the patient was lost to follow-up, and we were unable to ascertain the outcome of her treatment decision.

## Discussion

Based on the histopathological report, we considered a diagnosis of Ota nevus. It is a benign condition characterized by blue or dark pigmentation, appearing as violet or tan patches on the skin around the eyes and face. It was first described by Japanese dermatologist Ota in 1939 [3]. A distinctive feature is the involvement of the sclera on the same side as the affected skin, observed in approximately two-thirds of patients. The prevalence of Ota nevus is about 0.22% among Asians, and it is rarely seen in other populations [3,4], although it has been reported in Latin populations [5]. According to Tanino's traditional classification (IV) and PUMCH's (Peking Union Medical College Hospital) new classification (IVa), the bilateral subtype is seen in approximately 5% of cases [3]. Due to the rarity of this condition in our population and the bilateral presentation, we decided to reevaluate the case.

In the differential diagnosis, other causes of FM, such as melasma, Riehl's melanosis, lichen planus pigmentosus, erythema dyschromicum perstans, erythrosis, and poikiloderma of Civatte, were easily ruled out due to the mucosal and scleral involvement [1]. Given the scleral involvement, we considered dermal melanocytoses, such as acquired bilateral nevus of Ota-like macules (ABNOM) or Hori's nevus, which more commonly affect the face and are acquired conditions. Hori's nevus, first described by Hori et al. in 1984 [2,3], has a prevalence of 2.5% in the Asian population and is similar in appearance to Ota nevus, although it is more commonly bilateral [4]. Histologically, Hori's nevus is characterized by irregularly shaped, bipolar melanocytes dispersed in the papillary and mid dermis, particularly in the subpapillary dermis, forming perivascular clusters along with melanophages while maintaining normal skin architecture. In contrast, in Ota nevus, melanocytes are evenly and diffusely distributed throughout the papillary and reticular dermis [2,6].

Upon reviewing the case, we found that the melanocytes were not scattered throughout the dermis as described in the histopathological description but clustered in groups and dispersed perivascularly, with the presence of melanophages. Based on these histopathological findings and the bilateral lesions, we leaned toward ABNOM. However, our literature search revealed almost no reported cases in this age group. In a large study of 8,680 subjects with ABNOM, only one patient was under 18 years old [4], and there were no reported cases of scleral involvement in ABNOM. Interestingly, we found a study from a different state in China that included 46 pediatric patients, challenging the notion that ABNOM does not occur in childhood [7]. Additionally, we found the first reported case of conjunctival and oral mucosal involvement in ABNOM, described as a “new variant,” suggesting that previous cases of mucosal involvement might have been overlooked [8].

To the best of our knowledge, there are no other reported cases of ABNOM in Hispanic or Indigenous populations, and only one case of ABNOM affecting the sclera has been reported. We believe that, based on recent studies involving childhood cases, this condition is not limited to adulthood and may be underdiagnosed in non-Asian populations as well as in cases with mucosal involvement. Correct classification is crucial not only for epidemiological reasons but also because treatment responses to Q-switched laser therapy may differ between Ota nevus and ABNOM. Additionally, Ota nevus is better documented in terms of melanoma risk in the skin, eyes, and central nervous system, as well as the risk of glaucoma when ocular involvement is present [9]. The risk of glaucoma and ocular melanoma in ABNOM has not been reported.

The pathogenesis of both Ota nevus and ABNOM remains unknown, though a few theories have been proposed. A two-hit model suggests an initial migration to the skin followed by a melanocytosis trigger [2]. Cellular senescence is a state of irreversible growth arrest that occurs when cells can no longer divide. This process can be triggered by various factors, including the activation of oncogenes-genes that, when mutated or overexpressed, can drive cancer progression. Oncogene-induced senescence results from the hyperactivation of these oncogenic signaling pathways, which can lead to cellular growth arrest [10].

**Figure 3:**
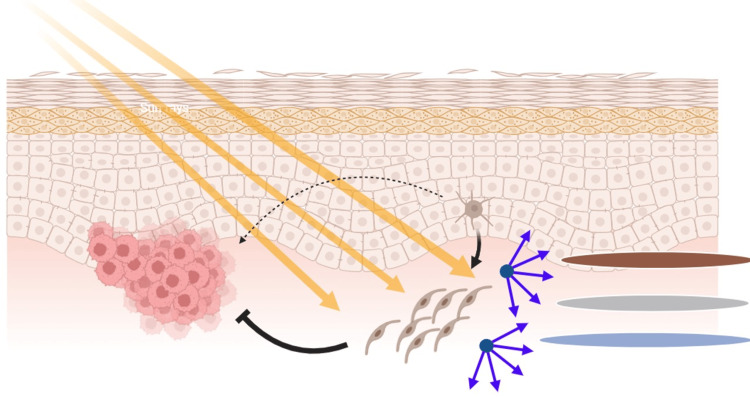
Melanocyte senescence hypothesis and pigmentation The image illustrates the hypothesis that melanocytes enter a senescent phenotype, maintaining metabolic activity and melanosome production while being in cell cycle arrest. This senescence acts as a defense mechanism against oncogene mutations to prevent oncogenesis. The depth of the clusters results in different shades of coloration, from grey to blue or brown, caused by the Tyndall effect (scattering of light by dermal particles, making deeper pigments appear blue) Image credits: Jesus Martinez MD. Created with biorender.com

In the context of Ota nevus and ABNOM, melanocytes may acquire mutations in oncogenes, such as GNAQ (Guanine Nucleotide Binding Protein, Q Polypeptide) [9], and enter this state of irreversible growth arrest. Although senescent cells cease to divide, they often maintain active metabolism and exhibit distinctive morphological changes [10]. This senescent state may account for the unique bipolar and fusiform shapes of melanocytes observed in Ota nevus and ABNOM, as well as the presence of melanosomes in various stages, reflecting ongoing metabolic activity [2]. Thus, the pigmentation and cellular patterns seen in Ota nevus and ABNOM may be linked to this senescent state and its associated metabolic processes. Further molecular studies are necessary to investigate this hypothesis and confirm these findings (Figure 3).

## Conclusions

Accurate classification of facial melanoses, such as Ota nevus and ABNOM, is essential for both epidemiological accuracy and informed treatment decisions, especially given the differing therapeutic responses and associated risks, such as the higher documented risk of melanoma and glaucoma in Ota nevus. Our case underscores the diagnostic challenges posed by these conditions, particularly in populations in whom they are less commonly reported. The need for increased awareness and consideration of ABNOM in non-Asian and pediatric populations is evident, as misclassification can lead to suboptimal management.

Further molecular studies are necessary to elucidate the pathogenesis of both Ota nevus and ABNOM, with a focus on the potential roles of cancer-associated mutations and cellular senescence. Such research could provide valuable insights into their etiology and inform more effective treatment strategies. Also, long-term studies on the ocular risks associated with ABNOM are warranted to determine if similar precautions as those for Ota nevus should be applied. Better understanding and proper classification of these dermal melanocytoses will improve patient outcomes via more accurate diagnoses, better-targeted treatments, and comprehensive risk management.
